# Renal Ischemia-Reperfusion Injury in a Diabetic Monkey Model and Therapeutic Testing of Human Bone Marrow-Derived Mesenchymal Stem Cells

**DOI:** 10.1155/2018/5182606

**Published:** 2018-08-01

**Authors:** Kyo Won Lee, Tae Min Kim, Kyeong Sik Kim, Seunghwan Lee, Junhun Cho, Jae Berm Park, Ghee Young Kwon, Sung Joo Kim

**Affiliations:** ^1^Department of Surgery, Division of Transplantation, Samsung Medical Center, Sungkyunkwan University School of Medicine, 81 Irwon-ro, Gangnam-gu, Seoul 06351, Republic of Korea; ^2^Graduate School of International Agricultural Technology and Institute of Green-Bio Science and Technology, Seoul National University, 1447 Pyeongchang-daero, Pyeongchang, Gangwon-do 25354, Republic of Korea; ^3^Department of Surgery, Kyung Hee University School of Medicine, Kyung Hee University Hospital at Gangdong, Seoul, Republic of Korea; ^4^Department of Pathology, Samsung Medical Center, Sungkyunkwan University School of Medicine, 81 Irwon-ro, Gangnam-gu, Seoul 06351, Republic of Korea

## Abstract

Clinically, acute kidney injury (AKI) episodes in diabetes mellitus (DM) patients are associated with a cumulative risk of developing end-stage renal disease. In this study, we asked whether the severity of AKI induced by renal ischemia-reperfusion injury (IRI) is more prominent in DM than in non-DM control using a cynomolgus monkey (*Macaca fascicularis)* model. We also investigated whether human bone marrow-derived mesenchymal stem cells (hBM-MSCs) infused via the renal artery could ameliorate renal IRI in DM monkeys. The experimental data, including mortality rate, histologic findings, and urinary albumin secretion indicate that the severity of AKI was greater in DM monkeys than in control animals. Moreover, histological findings and qRT-PCR analysis of *Ngal* mRNA in renal biopsy tissue showed that hBM-MSC promoted the recovery of tubular damage caused by AKI. Serum analysis also revealed that the level of albumin and ALT was increased 24 and 48 hours after AKI, respectively, suggesting that AKI induced acute liver injury. We suggest that this nonhuman primate model could provide essential information about the renal and nonrenal impairment related to DM and help determine the clinical usefulness of MSCs in AKI.

## 1. Introduction

Prolonged hyperglycemia causes various renal stress responses, and it is well reported that diabetes is the single largest contributor to the growing prevalence of chronic kidney disease (CKD) [1, 2]. On the other hand, acute kidney injury (AKI) is a disorder characterized by a rapid decrease in renal function, and it is understood to be associated with renal and systemic inflammation [3]. Although diabetes mellitus (DM) and AKI are separate diseases with distinct pathophysiologies, recent studies have demonstrated their interdependent relationship [4, 5]. Indeed, episodes of AKI in diabetic patients are associated with a cumulative risk of developing end-stage CKD [6]. Specifically, a meta-analysis showed that preadmission DM status, arterial hypertension, and proteinuria are independent AKI risk factors [7]. Also, preclinical studies showed that diabetes can increase the susceptibility of AKI [8–11].

During the last several decades, mesenchymal stem cells (MSCs) have emerged as an innovative tool for tissue regeneration therapy, mainly due to its unique secretome profile and capacity for self-renewal and differentiation [12]. Their reparative ability on damaged tissue are mainly attributed to paracrine effects, transdifferentiation, and the release of extracellular vesicles [13], and numerous evidence in rodent models have revealed the therapeutic potential of human MSCs in various models of tissue repair including AKI [14].

Many studies have shown that inflammatory responses, including those involved in AKI, differ between humans and mice [15–17], and a recent finding demonstrated that the renal structure also differs between humans and mice [18]. Thus, using animal models other than rodents could better translate preclinical findings into the human setting. In this study, we developed a nonhuman primate model of renal ischemia-reperfusion injury (IRI), which is the most common cause of AKI in human [19]. We used this model to confirm whether diabetes can increase the susceptibility to AKI and also to evaluate the efficacy of human bone marrow-derived MSCs (hBM-MSCs) in reducing renal IRI.

## 2. Materials and Methods

### 2.1. Animals

Male, 5- to 8-year old cynomolgus monkeys (*Macaca fascicularis*) weighing between 2.8 and 5 kg were used in this study. All animals were originated from Cambodia. The animal procedures were conducted in accordance with the *Guide for the Care and Use of Laboratory Animals* [20] and the Animal Welfare Act [21] in the animal facility of the Primate Organ Transplantation Research Center at Genia (Sung-nam City, Korea). Animals were individually housed indoors on a 12 : 12 h, light–dark cycle and were fed standard macaque biscuits (Certified Primate Diet 5048^∗^, LabDiet, St. Louis, MO, USA) and fresh fruits twice daily. Animal rooms were maintained at 23 to 25°C and 40% to 60% relative humidity, with 15 changes of conditioned air hourly. Chlorinated, filtered fresh water was provided without restriction. Tuberculosis was tested every six months with immunochromatographic test kit (SD Bioline TB Ag MPT64 RAPID, Standard Diagnostics, Yongin-si, South Korea), and all were negative during the study period. Tests on herpes B virus, simian T-cell leukemia virus, simian retrovirus, simian immunodeficiency virus, measles, cynomolgus cytomegalovirus, and simian varicella virus were conducted at a diagnostic laboratory (Zoologix Inc., CA), and all were diagnosed negative. This animal study was approved by the Institutional Animal Care and Use Committee of Orient Bio Laboratories (ORIENT–IACUC–16317).

### 2.2. Induction and Management of Diabetes Mellitus

DM was induced by subtotal pancreatectomy and a 60 mg/kg injection of streptozotocin (STZ, Sigma, St. Louis, MO, USA). Subjects did not receive food or liquid for 12 h prior to surgery. After intramuscular injection of 10 mg/kg ketamine, subjects were intubated with 4.0 to 4.5 Fr endotracheal tubes, and general anesthesia was induced with 3–5% isoflurane. Subjects were kept under anesthesia with 1-2% isoflurane, nitrous oxide, and oxygen. Prophylactic antibiotics (20 mg/kg cefazolin sodium intravenously) were given at the time of skin incision. STZ was given intravenously immediately after the pancreatectomy.

Type 1 DM (T1DM) was diagnosed upon satisfaction of all of the following criteria: (1) sustained hyperglycemia (blood glucose level > 250 mg/dl), (2) fasting NHP C-peptide level below 0.5 ng/ml or less than one-third of preinduction levels, and (3) absence of stimulated C-peptide response with intravenous glucose tolerance test (IVGTT). We measured serum C-peptide using a radioimmunoassay kit developed for human plasma (C-peptide IRMA kit; IMMUNOTECH, Beckman Coulter Inc., Prague, Czech Republic), which shows 90% cross-reactivity with cynomolgus monkeys. IVGTT was measured after 12 hours of fasting. After sedation with ketamine, three blood samples were drawn for C-peptide and blood glucose measurements. Then 0.5 g/kg dextrose was given intravenously, and blood samples were drawn 1, 3, 5, 7, and 10 min thereafter. Blood samples were drawn at 15, 20, 25, 30, and 60 min to measure the glucose disappearance rate. Acute C-peptide response (ACR) was calculated as the difference between mean C-peptide after glucose infusion and C-peptide at baseline. The glucose disappearance rate (K_G_, %/min) was calculated as the slope of the decline in the log-transformed value of blood glucose between 10 and 30 min.

After successful induction of T1DM, blood glucose levels were checked 3 to 4 times daily and maintained between 200 and 300 mg/dl with insulin (glargine (Lantus^®^, Sanofi-Aventis, Bridgewater, NJ, USA) and glulisine (Apidra^®^, Sanofi-Aventis)). Body weight measurements and physical examinations were performed at regular intervals. The following blood hematological parameters were also checked regularly: white blood cell count (WBC) with differential count, hemoglobin (Hb), hematocrit and platelet count, aspartate aminotransferase (AST), alanine aminotransferase (ALT), alkaline phosphatase (ALP), total bilirubin, blood urea nitrogen (BUN), creatinine, albumin, globulin, sodium, potassium, chloride, calcium, inorganic phosphorus, cholesterol, triglyceride, amylase, and C-reactive protein (CRP). The fasting glucose levels of non-DM monkeys were less than 100 mg/dl in all cases and those of DM monkeys were more than 250 mg/dl in all cases at least for 5 months prior to this experiment.

### 2.3. Human Bone Marrow-Derived Mesenchymal Stem Cell Preparation

HBM-MSCs were obtained from the Catholic Institute of Cell Therapy (CIC; Seoul, Korea). Human bone marrow aspirates were obtained from the iliac crest of healthy donors aged 20 to 55 years after approval by the Institutional Review Board of Seoul St. Mary's Hospital. Bone marrow aspirates were obtained from healthy donors after written informed consent and sent to the Good Manufacturing Practice-compliant facility of the CIC. The detailed procedures for the isolation, expansion, and quality control of MSCs, including differentiation potential (adipogenic, chondrogenic, and osteogenic differentiation) and cell surface marker analyses, are described previously [22].

### 2.4. Renal Ischemia-Reperfusion Injury Model and Experimental Design

We allocated eight monkeys into three groups (*N* = 3, 3, and 2 in Groups 1, 2, and 3, resp.). Three normal animals (Group 1) and five DM animals were subjected to renal IRI for one hour, and 2 of the latter 5 received 5 × 10^6^ cell/kg of hBM-MSCs by injection (Group 3). To induce IRI, the monkeys were anesthetized, and midline incisions were made. The renal pedicles were exposed and subsequently clamped with bulldog clamps for one hour (Supplementary Figure 1). In Group 3, 5 × 10^6^ cell/kg of hBM-MSCs was injected into the suprarenal aorta after reperfusion while the aorta was clamped just below the renal arteries to allow blood flow only to the kidneys. Blood and urine samples were obtained, and a kidney gun biopsy from each kidney was performed every 24 hours after reperfusion.

### 2.5. Assessment of Renal Injury

We performed serum biochemical analysis of Cr, BUN, CRP, and total protein levels (FUJI DRI-CHEM 4000i, Tokyo, Japan). The concentration of interferon gamma (IFN-*γ*) and tumor growth factor alpha (TGF-*α*) in serum were detected using Luminex assay kits (EMD Millipore, Billerica, MA) and then normalized against the amount of urine creatinine. Albumin secretion in urine was measured by monkey albumin ELISA kit (Abcam, Cambridge, UK). The concentration of urinary neutrophil gelatinase associated lipocalin (NGAL) at each designated time point was analyzed using a monkey NGAL ELISA kit (Bioporto, Hellerup, Denmark).

At designated time points after renal IRI (0, 24 and 48 hours), renal biopsy slices were obtained from the right kidney, fixed in 10% formalin, and embedded in paraffin wax. Tissue sections of 5 *μ*m thickness were stained with hematoxylin and eosin (H&E) and examined under a light microscope to discern any histological changes. To morphologically assess the extent of tubular injury and necrosis, H&E stained sections were reviewed by a renal pathologist who was blinded to the experimental groups. The proportion of areas with tubular injury or necrosis to areas with relatively intact tubules was visually estimated. Tubular injury was identified in areas with cytoplasmic vacuolization, loss of brush border, flattening of epithelium, or evidence of tubular necrosis as manifested by granular casts and necrotic debris.

Total RNA was isolated using the TRIzol^®^ method from snap-frozen renal biopsy tissues collected from the left kidney (Sigma Aldrich, St. Louis, MO). cDNA synthesis and quantitative real time reverse transcription PCR (qRT-PCR) were conducted as previously described [23]. Primer sequences were as follows: *Gapdh*, forward: 5′-CGGAGCTCTCCAGAACATCA-3′, reverse: 5′-ggtcaggtccaccactgaca-3′; *Ngal*, forward: 5′-ctgtcagggaatgcagttgg-3′, reverse: 5′-caggatggaggtcacgttgt-3′.

### 2.6. Statistical Analysis

Differences among the three groups at specific time points were analyzed using the Kruskal-Wallis test. Repeated ANOVA was applied to repeated measurements of parameters. The statistical significance between two groups was determined using two-tailed unpaired *t*-tests (tubular injury extent between non-DM and DM animals and qRT-PCR of Ngal mRNA). *P* values < 0.05 were considered significant and were corrected using Bonferroni's method in post hoc analyses. Statistical analyses were performed using SPSS version 22.0 (IBM, Armonk, NY, USA). The datasets analyzed during the current study are available from the corresponding author on reasonable request.

## 3. Results and Discussion

In this study, we investigated whether diabetes aggravates the severity of renal IRI-induced AKI in cynomolgus monkeys and assessed the therapeutic potential of intra-arterially injected hBM-MSCs. Although renal IRI models normally require a pilot study to determine the ischemia duration [24], we used a one-hour bilateral warm ischemic injury model based on the results of our pilot studies for renal transplantation in monkeys (data not included). The number of MSCs for intra-arterial injection was based on the previous study that had assessed the effect of MSCs in an interstitial fibrosis model using rhesus monkeys [25]. To ensure successful modeling, we monitored changes in the kidneys during the ischemia period (Supplementary Figure 1).  Table 1 shows that this protocol was tolerable in normal monkeys but not in DM monkeys; all the non-DM monkeys (Group 1) survived until day 4 after AKI, whereas the DM animals (Group 2) died within 60 hours. Of the two DM monkeys that received hBM-MSCs (Group 3), one monkey died after 72 hours and the other 53 hours. In Groups 2 and 3, the monkeys showed respiratory distress symptoms before they died. When they were examined by autopsy, pleural effusion was detected in common, while their kidneys were not ischemic in gross appearance. Microscopically, we found a large extent of necrosis and fluid retention in the tubules, while liver tissues showed hepatocyte swelling and fatty change. Also, alveolar capillary interstitial edema was detected in the lung (Supplementary Figures 2 and 3). From these results, we suspected that fluid overloading due to severe AKI as well as multiorgan (i.e., the lung and liver) damage secondary to AKI may have contributed to early death in DM animals. In group 3, we could not see any clinical evidence of rejection response against hBM-MSCs such as hypotension or fever immediate after injection.

**Figure 1:**
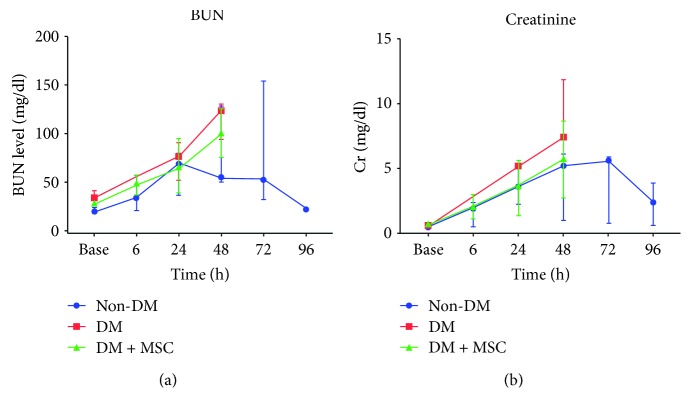
The survival rate and changes in renal function markers. DM monkeys that experienced renal ischemia-reperfusion injury (IRI) were subsequently injected with MSC (DM + MSC) or not (DM). Non-DM animals were used as the control. The serum levels of creatinine (a) and blood urea nitrogen (BUN) (b) were measured at designated study points. Data are median with range. *n* = 3, 3, and 2 in the non-DM, DM, and DM + MSC groups, respectively.

Figure 1(a) shows that the BUN level of the control animals peaked at 24 hours and then decreased thereafter, whereas that of the DM monkeys kept increasing for 48 hours. MSC treatment of the DM animals led to a partial decrease at 48 hours. The serum creatinine level tended to increase more abruptly in the DM animals than in normal animals, while MSC treatment partly contributed to lowering its level in DM monkeys (Figure 1(b)). Thus, our data indicate that DM compromised the monkey's ability to recover from AKI, and that the effect of MSCs on AKI recovery was not clear based on the results of the renal function marker during the study period.

**Figure 2:**
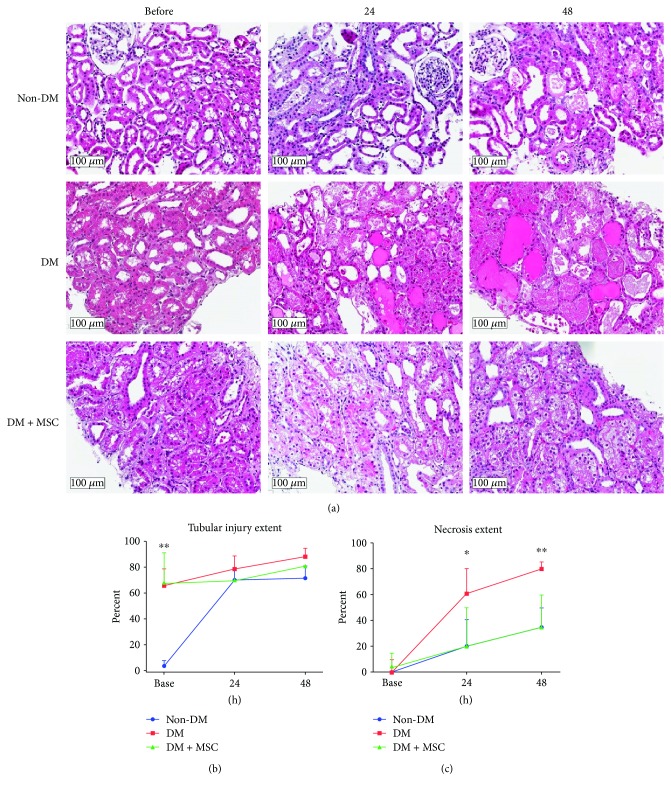
Histological examination of renal tissues. At designated time points, renal biopsy specimens were collected and subjected to H&E staining (a) to assess their tissue morphology. Scale bar = 100 *μ*m. Magnification: ×200. The extent of tubular injury (b) and necrosis (c) were also calculated. *n* = 3, 3, and 2 in the non-DM, DM, and DM + MSC groups, respectively. ^∗^ *P* < 0.05 and ^∗∗^ *P* < 0.001.

To further investigate the effect of DM on AKI progression, a renal pathologist who was blinded to the experimental groups conducted histological analyses on renal biopsies from the animals. As shown in Figure 2(a), a diverse pattern of tubular injury, such as cytoplasmic vacuolization, loss of brush border, flattening of epithelium, and luminal cell debris, was seen after IRI. Histologic findings showed preexisting tubular injury before IRI in DM animals (*P* < 0.001, Figure 2(b)), while the degree of tubular injury at 24 and 48 hours in DM animals was comparable to those of non-DM monkeys after ischemic insult (Figures 2(a) and 2(b)). The coagulation necrosis of renal tubules and prominent hyaline casts was also more evident postischemia in the DM group compared to the control group (Figure 2(c)). In DM animals treated with MSCs, the gross morphologies at 48 hours appeared to resolve better than those seen in the noninjected DM animals (Figure 2(a)). Similarly, among DM animals, a reduction in tubular necrosis was found in the MSC-treated group at 24 (*P* < 0.05) and 48 hours (*P* < 0.001, Figure 2(c)). Thus, hBM-MSC treatment apparently led to tubular regeneration within 24–48 hours, and those changes might have preceded functional recovery as shown by the serum markers (Figures 1(a) and 1(b)).

**Figure 3:**
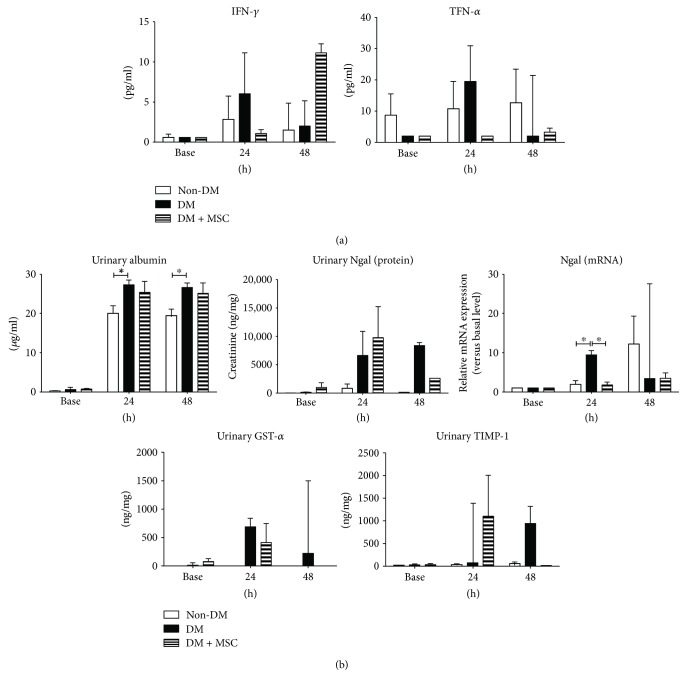
Analysis of proinflammatory markers following renal IRI and MSC treatment. (a) Blood was collected every 24 hours, and the serum levels of each cytokine were measured by a multiplex bead assay. Data are median with range. (b) Analysis of urinary markers for AKI. The concentration of urinary albumin and NGAL was measured by ELISA, and *Ngal* mRNA in renal tissue was analyzed by qRT-PCR. The level of GST-alpha and TIMP-1 in urine was obtained by a multiplex bead assay. Data are the median with range. *n* = 3, 3, and 2 in the non-DM, DM, and DM + MSC groups, respectively. ^∗^ *P* < 0.05.

The cytokine analysis shows that the serum concentrations of IFN-*γ* and TNF-*α*, which are known mediators of AKI progression [26, 27], were not different among groups at 24 hours. Unlike the tissue recovery by MSC treatment at 48 hours, their level seems to be even higher than those from nontreated animals. These finding possibly suggests that the utilization of these two early inflammatory markers may not be an ideal option for evaluating early progress of AKI in monkeys. (Figure 3(a)). Urine AKI markers were also analyzed and found a significant increase of albumin at 24 and 48 hours in DM compared with non-DM monkeys (*P* < 0.05, Figure 3(b)). We also found out that the level of urinary NGAL protein, which is also an AKI marker, was similarly detected among groups at 24 and 48 hours (Figure 3(b)). However, qRT-PCR showed that the expression of *Ngal* mRNA was significantly higher in the renal tissue of DM animals 24 hours after IRI compared with non-DM monkeys, and hBM-MSC treatment decreased its expression to a level comparable to that seen in the non-DM control (*P* < 0.05). We also measured the level of GST-alpha and TIMP-1, which are potential AKI biomarkers in urine [28, 29], and found out that the increase of GST-alpha was more evident in DM animals than in non-DM animals at 24 and 48 hours. A similar pattern was observed in the TIMP-1 level only at 48 hours. From these three urinary proteins, we could not observe their remarkable changes after MSC treatment. Based on these findings, we suggest that DM animals tend to be more susceptible to early ischemic injury as shown by urinary AKI marker secretion, and that MSC treatment was not effective in renal recovery at least during 48 hours after AKI.

Recently, the mechanism by which AKI triggers more severe diseases in DM has been studied in rodent models. Consistent with our results, Gao et al. [9] demonstrated that the AKI sensitivity of diabetic mice could be suppressed using a TNF-*α* neutralizing antibody, thereby supporting the role of the TNF-*α*-related inflammatory response in AKI sensitivity. Another study suggested that renal failure manifested by chronic inflammation and vasculopathy could be accelerated by postischemic inflammation in obese-diabetic rats [8].

**Figure 4:**
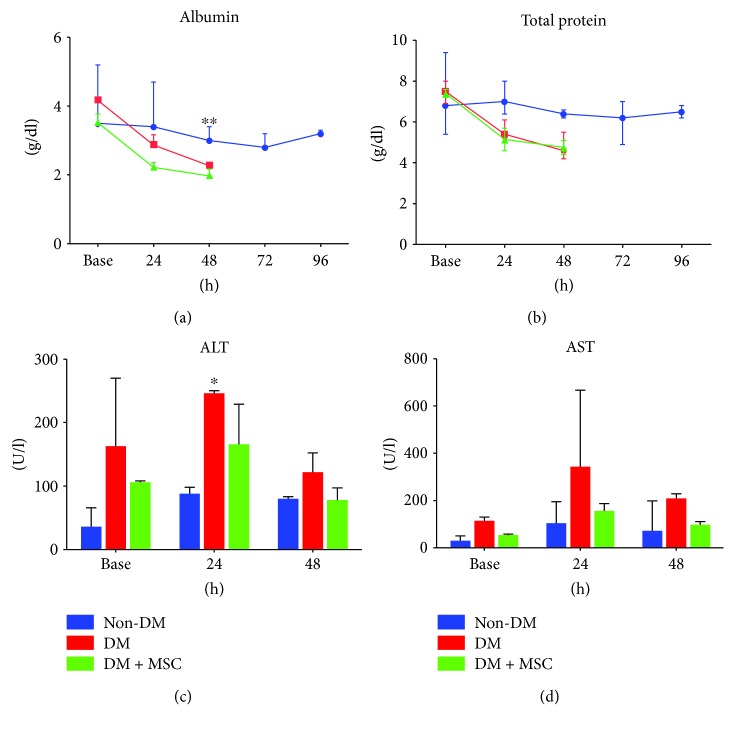
Serum biochemical analysis of markers indicative of liver function. Blood was collected every 24 hours, and the serum levels of total protein (a), albumin (b), ALT (c), and AST (d) were measured. *n* = 3, 3, and 2 in the non-DM, DM, and DM + MSC groups, respectively. ^∗^ *P* < 0.05 and ^∗∗^ *P* < 0.01. Data are the mean with range.

The serum levels of albumin and total protein declined as AKI progressed, and a significant decrease in albumin was found in DM animals at 48 hours, compared with non-DM monkeys (Figure 4(a), *P* < 0.05). A similar pattern of down-shift was detected in the total protein level, though that difference was not statistically significant (Figure 4(b)). These results indirectly suggest that AKI might have caused an injury to the livers of DM animals, because the liver is the major organ that synthesizes and secretes albumin and immunoglobulins [30]. In line with this, the ALT serum level was significantly higher in the DM animals than in the DM + MSC and non-DM animals at 24 hours (Figure 4(c), *P* < 0.05), though no difference was observed at 48 hours. In contrast, no significant difference was found in AST levels among the groups, possibly due to the large within-group variances caused by the small number of animals (Figure 4(c)). Thus, we suggest that AKI might have led to an abrupt injury to the liver. Liver tissues at the end of the experiment showed hepatocyte swelling and fatty change. Those findings were observed more severely in the DM animals (Supplementary Figure 2). Also, inflammatory cell infiltration was observed in the lung tissue of Groups 1 and 2 animals during necropsy (Supplementary Figure 3). Indeed, previous studies have shown that AKI can induce secondary injuries to other organs, including the liver and lungs—the so-called organ cross talk [31, 32].

Autologous or allogenic MSC-based therapy has been suggested as an alternative treatment for AKI [33], and numerous rodent studies reported that systemic injection of human MSCs contributed to renal function recovery by various processes such as regulating immune cell trafficking, reducing oxidative stress, stimulating tubular proliferation, or differentiating into tubular epithelial cells [25, 34–39]. In most cases of rodent studies, systemic delivery of human BM-MSCs has been widely used to study their therapeutic potential mainly due to the easiness in cell injection and future clinical application. However, this method has several disadvantages in that injected cells can be accumulated in the organs including the lung and liver [40]. Also, the cells can be diluted via systemic circulation. Accordingly, the intra-arterial injection has been attempted in several kidney diseases using large animal models including ewe and rhesus monkeys [25, 41]. Based on the methods that these studies had used, we adopted an alternative route whereby the suprarenal aorta was used while the infra-renal aorta was occluded to allow the MSCs to naturally go into the kidneys via downstream of the renal artery because direct injection into the renal artery was unavailable due to its small diameter (<1 mm) and the risk of bleeding. We consider that the renal or systemic effect of occlusion of the infra-renal aorta was minimal because the overall time for the cell administration took less than a minute.

Our renal IRI protocol in DM monkeys might not be optimal. Apparently, the duration of renal pedicle clamping in our protocol provoked severe AKI for DM monkeys; therefore, the duration should be reduced so that long-term follow-up data with less variance can be obtained. Although histological studies supported our hypothesis, mortality as early as 49–60 hours in DM animals did not enable us to further monitor decreases in the BUN or creatinine of DM animals. Also, it should be noted that we used only a few monkeys in our experiments, which might have contributed to significant intra- or intergroup variations. The potential effects of other intrinsic/extrinsic factors, such as differences in diabetic condition or subtle changes in procedures during AKI induction, should also be noted. Nonetheless, our finding will provide important aspects while conducting experiments that therapeutic cell or biomolecules are tested for reducing AKI using nonhuman primates. This model will also be beneficial in developing new therapies to ameliorate AKI that occurs during organ transplant.

## 4. Conclusions

Using a nonhuman primate model, we showed that DM increases susceptibility to renal ischemia-reperfusion injury, and that hBM-MSCs have the potential to attenuate renal injuries.

## Figures and Tables

**Table 1:** Allocation of animals and survival rate in each group.

Group	Without (−) or with (+) DM	MSC treatment	Number of animals	Hours survived by each animal
1	(−)	(−)	3	>96, >96, >96
2	(+)	(−)	3	60, 53, 49
3	(+)	(+)	2	72, 53

## Data Availability

The data used to support the findings of this study are available from the corresponding author upon request.

## Supplementary Materials

Supplementary Materials Supplementary Figure 1: representative images of inducing renal ischemia and reperfusion injury by the exposure and occlusion of the renal pedicles. A uniform dusky color appeared in both the right (a) and left (b) kidneys after bulldog clamps were applied to the renal pedicles. After 1 hour, the clamps were removed, and the reversal of the dusky appearance was confirmed in both the right (c) and left (d) kidneys. Supplementary Figure 2: histologic examination of liver tissues with H&E staining. Non-DM monkeys (a), DM monkeys (b), and DM + MSC monkeys (c) at the end of the experiment. Hepatocyte swelling and fatty change were observed in one of three non-DM monkeys (a). However, those findings were observed more severely in all of DM monkeys (b) and MD + MSC monkeys (c). Scale bar = 100 *μ*m. Magnification: ×200. Supplementary Figure 3: histologic examination of lung tissues with H&E staining. Non-DM monkeys (a), DM monkeys (b), and DM + MSC monkeys (c) at the end of the experiment. Interstitial inflammatory cell infiltration was observed in one of three non-DM monkeys (a). Alveolar capillary interstitial edema and interstitial inflammatory cell infiltration were observed in one of three DM monkeys (b). Alveolar capillary interstitial edema was observed in one of two DM + MSC monkeys (c). Scale bar = 100 *μ*m. Magnification: ×200.
