# Supplementary material for: Renal Ischemia-Reperfusion Injury in a Diabetic Monkey Model and Therapeutic Testing of Human Bone Marrow-Derived Mesenchymal Stem Cells
Source: J Diabetes Res. 2018 Aug 1;2018:5182606. doi: 10.1155/2018/5182606 (PMC6092988; doi:10.1155/2018/5182606)
Supplement: Supplementary Materials — Supplementary Figure 1: representative images of inducing renal ischemia and reperfusion injury by the exposure and occlusion of the renal pedicles. A uniform dusky color appeared in both the right (a) and left (b) kidneys after bulldog clamps were applied to the renal pedicles. After 1 hour, the clamps were removed, and the reversal of the dusky appearance was confirmed in both the right (c) and left (d) kidneys. Supplementary Figure 2: histologic examination of liver tissues with H&E staining. Non-DM monkeys (a), DM monkeys (b), and DM + MSC monkeys (c) at the end of the experiment. Hepatocyte swelling and fatty change were observed in one of three non-DM monkeys (a). However, those findings were observed more severely in all of DM monkeys (b) and MD + MSC monkeys (c). Scale bar = 100 μm. Magnification: ×200. Supplementary Figure 3: histologic examination of lung tissues with H&E staining. Non-DM monkeys (a), DM monkeys (b), and DM + MSC monkeys (c) at the end of the experiment. Interstitial inflammatory cell infiltration was observed in one of three non-DM monkeys (a). Alveolar capillary interstitial edema and interstitial inflammatory cell infiltration were observed in one of three DM monkeys (b). Alveolar capillary interstitial edema was observed in one of two DM + MSC monkeys (c). Scale bar = 100 μm. Magnification: ×200. [file 5182606.f1.docx]

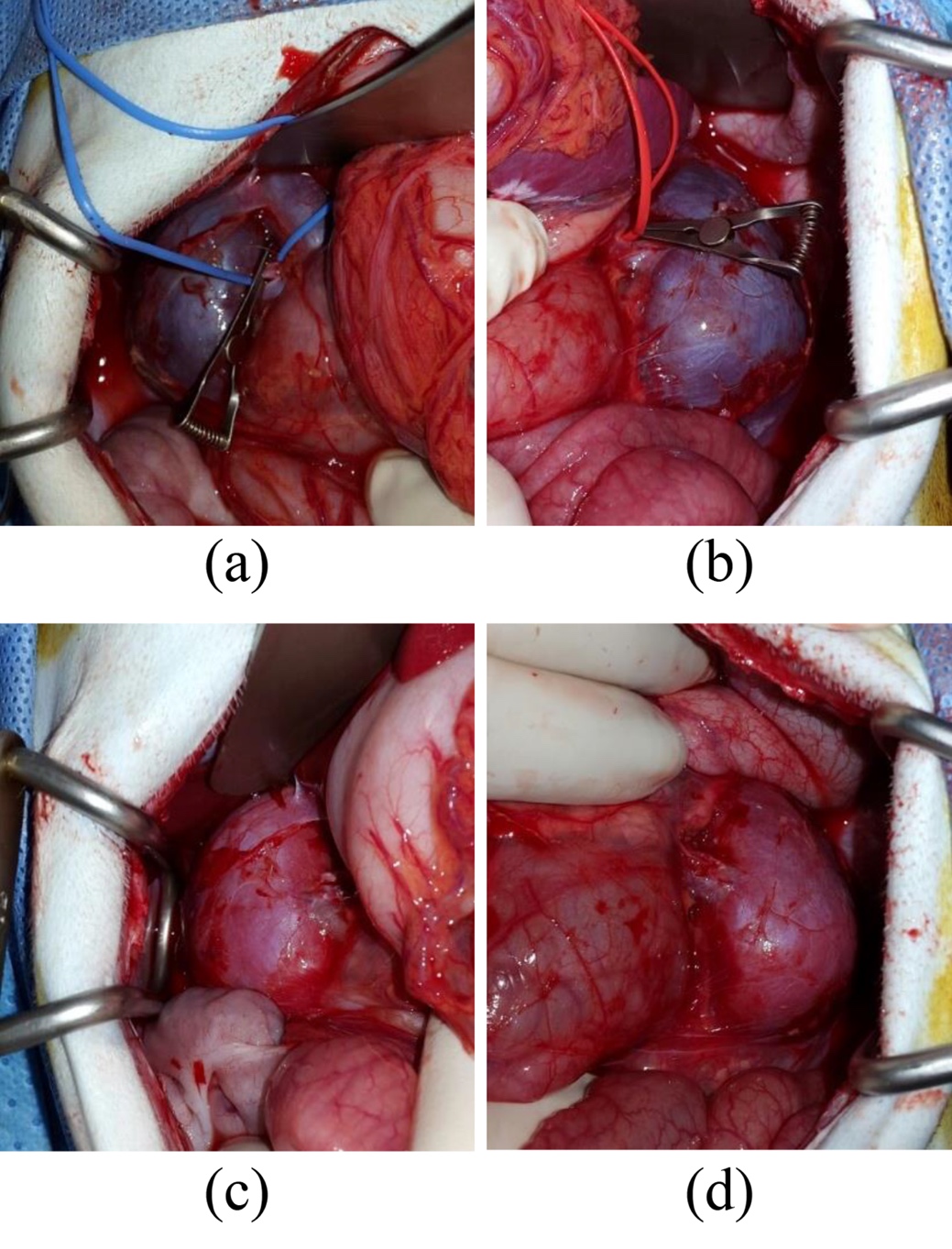


SupplEmentary Figure 1: Representative images of inducing renal ischemia and reperfusion injury by the exposure and occlusion of the renal pedicles. A uniform dusky color appeared in both the right (a) and left (b) kidneys after bulldog clamp were applied to the renal pedicles. After 1 hour, the clamps were removed, and the reversal of the dusky appearance was confirmed in both the right (c) and left (d) kidneys.


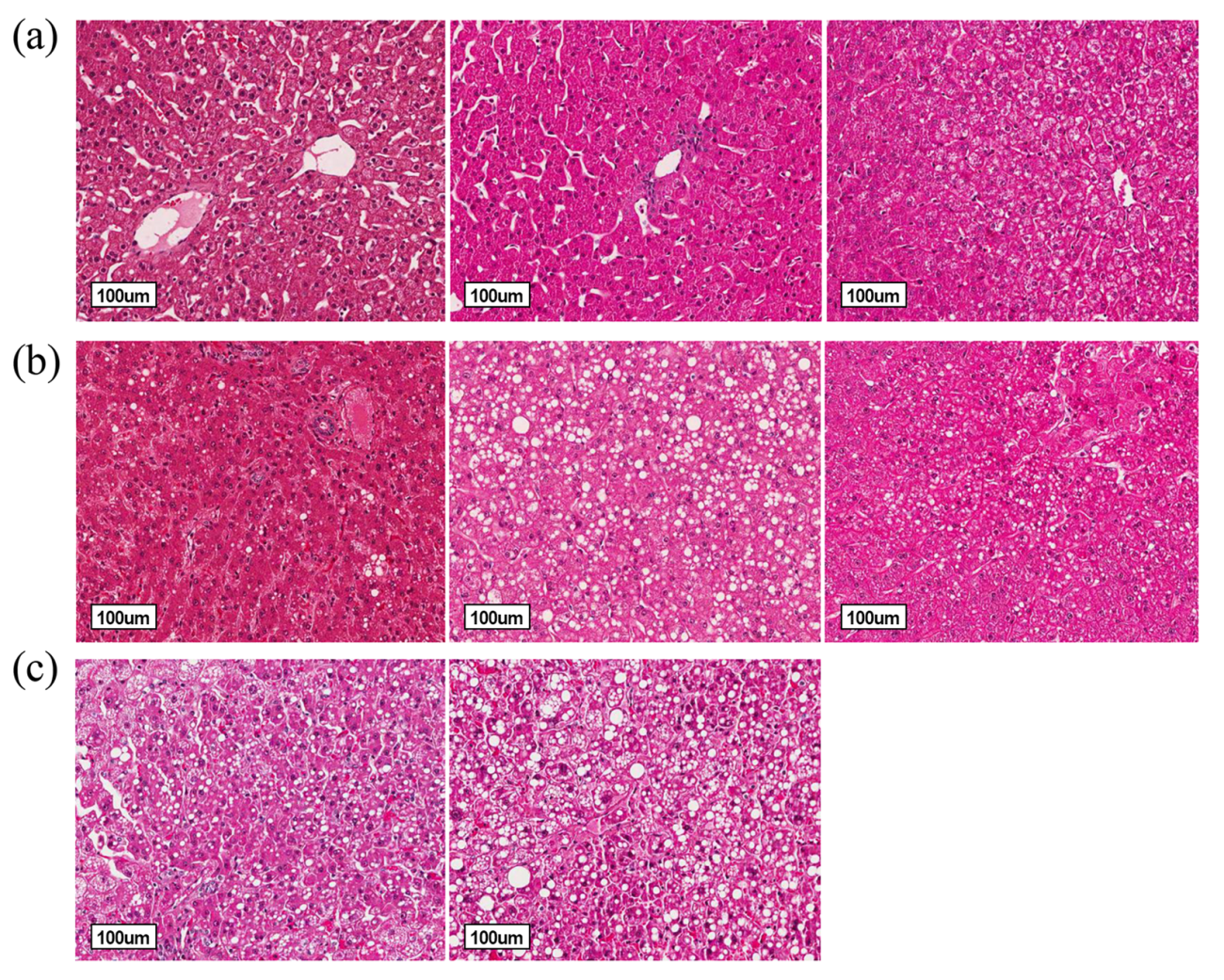


SupplEmentary Figure 2: Histologic examination of liver tissues with H&E staining. Non-DM monkeys (a), DM monkeys (b), and DM+MSC monkeys (c) at the end of the experiment. Hepatocyte swelling and fatty change were observed in one of three non-DM monkeys (a). However, those findings were observed more severely in all of DM monkeys and DM+MSC monkeys (b)(c). Scale bar = 100µm. Magnification: x200.


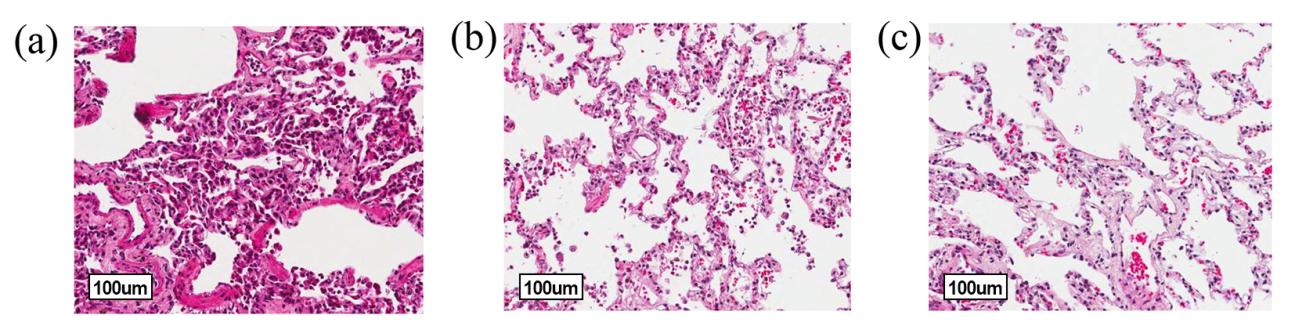


SUPPLEMENTARY FIGURE 3: Histologic examination of lung tissues with H&E staining. Non-DM monkeys (a), DM monkeys (b), and DM+MSC monkeys (c) at the end of the experiment. Interstitial inflammatory cell infiltration was observed in one of three non-DM monkeys (a). Alveolar capillary interstitial edema and interstitial inflammatory cell infiltration was observed in one of three DM monkeys (b). Alveolar capillary interstitial edema was observed in one of two DM+MSC monkeys (c). Scale bar = 100µm. Magnification: x200.
